# Majorana quasiparticles and topological phases in 3D active nematics

**DOI:** 10.1073/pnas.2405304121

**Published:** 2024-12-19

**Authors:** Louise C. Head, Giuseppe Negro, Livio N. Carenza, Nathan Johnson, Ryan R. Keogh, Giuseppe Gonnella, Alexander Morozov, Enzo Orlandini, Tyler N. Shendruk, Adriano Tiribocchi, Davide Marenduzzo

**Affiliations:** ^a^School of Physics and Astronomy, University of Edinburgh, Edinburgh EH9 3FD, United Kingdom; ^b^Physics Department, College of Sciences, Koç University, Sarıyer 34450, Istanbul, Türkiye; ^c^Dipartimento di Fisica, Università degli Studi di Bari and Istituto Nazionale di Fisica Nucleare (INFN), Bari I-70126, Italy; ^d^Department of Physics and Astronomy, University of Padova and INFN, Padova I-35131, Italy; ^e^Istituto per le Applicazioni del Calcolo, Consiglio Nazionale delle Ricerche, Roma 00185, Italy

**Keywords:** liquid crystals, disclinations, Majorana fermions, active nematics

## Abstract

Quasiparticles are low-energy excitations with important roles in condensed matter physics. An intriguing example is provided by Majorana quasiparticles, which are equivalent to their antiparticles. Despite being implicated in neutrino oscillations and topological superconductivity, their experimental realizations remain very rare. Here, we propose a purely classical realization of Majorana fermions in terms of three-dimensional disclination lines in active nematics. The underlying reason is the well-known equivalence, in 3D, between a +1/2 local defect profile and a −1/2 profile, which acts as its antiparticle. The mapping also requires proving that defect profiles transform as spinors, and activity is needed to overcome the elastic cost associated with these excitations, so they spontaneously appear in steady state. We combine topological considerations and numerics to show that active nematics under confinement spontaneously create in their interior topologically charged disclination lines and loops, akin to Majorana quasiparticles with finite momentum. Within a long channel, the phenomenology we observe resembles that of the Kitaev chain, as Majorana-like states appear near the boundaries, while a delocalized topological excitation arises in the form of a chiral disclination line. The analogy between 3D nematic defects and topological quasiparticles further suggests that active turbulence can be viewed as a topological phase, where defects percolate to form delocalized topological quasiparticles similar to those observed in the channel. We propose that three-dimensional active disclinations can be used to probe the physics of Majorana spinors at much larger scale than that for which they were originally introduced, potentially facilitating their experimental study.

## Significance

Majorana fermions are particles which are equivalent to their antiparticles. They were proposed long ago, but no elementary particle with their properties has yet been found. Instead, quantum condensed matter has provided examples of excited collective states in nanowires and superconductors which behave as Majorana quasiparticles. Here, building from the well-known fact that topological charge of nematic defects is only conserved modulo 2 in 3D, we show that a topologically charged disclination loop provides a classical analogue of a Majorana particle. Besides being of fundamental interest, this mapping suggests a different way to interpret active nematic turbulence, as a topological phase with delocalized quasiparticles. It may also allow probing Majorana physics and applications to topological computers at unexpectedly large lengthscales.

Liquid crystals have long served as a fruitful playground where abstract ideas from mathematics, condensed matter, cosmology, and particle physics can find a practical application (1–3). A well-known example is the topological theory of defects and disclinations in nematics and cholesterics in two and three dimensions, which provides a tangible application of homotopy theory (2, 4). Another instance is given by topological solitons, such as skyrmions, torons, and hopfions, which behave as quasiparticles with rich and fascinating emerging phase behavior and dynamics (5–10). With respect to other condensed matter systems such as superconductors and superfluids, which also harbor nontrivial topological phases and quasiparticles (11–14), the spatiotemporal scales of liquid crystal patterns are usually much larger—a fact that can be useful to simplify their experimental studies.

**Fig. 1.**
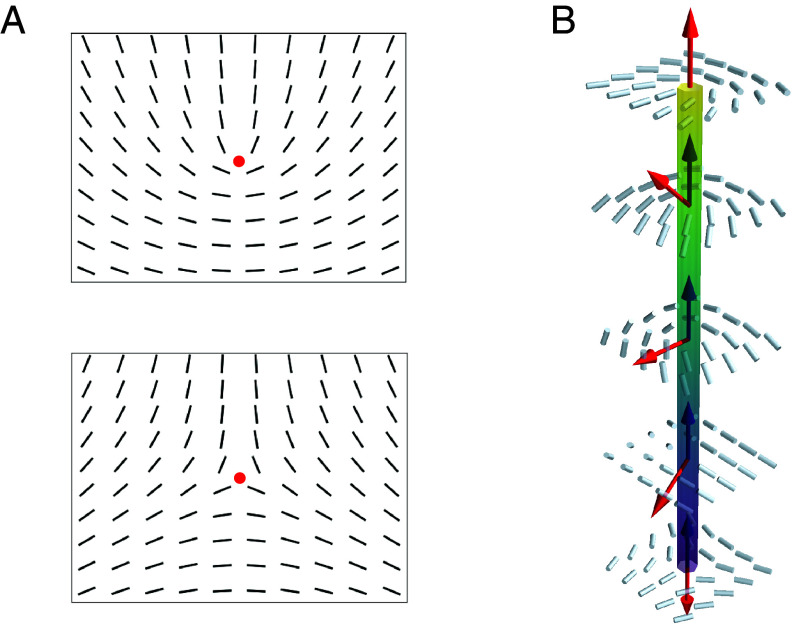
(*A*) Local planar director profile for topological charge s=1/2 (*Top*) and s=−1/2 (*Bottom*) disclinations, with the defect shown as a red dot. The two patterns are distinct in 2D, but they can be mapped onto each other when embedded in 3D. Either profile can be viewed as a Majorana quasiparticle at rest. (*B*) A disclination line containing a +1/2 and a −1/2 local defect profile, which is therefore equivalent to the trivial (defect-free) nematic state. This line can therefore be viewed as a pair of Majorana quasiparticles. The coloring indicates the value of cosβ=T·Ω, with T (corresponding to the blue arrows) and Ω (red arrows) respectively the local tangent and rotation vector (36–38) for each point on the disclination.

Here, we show that liquid crystals possess an intriguing qualitative analogue of Majorana excitations, which are quasiparticles that are equivalent to their corresponding antiparticles (15). Specifically, we show that the liquid crystal analogue of a Majorana particle is a three-dimensional and topologically charged nematic disclination loop. The analogy holds because a +1/2 topological defect profile can be smoothly transformed, in 3 dimensions, into a −1/2 profile (see, e.g., refs. 16 and 17 and Fig. 1). Additionally, a local nematic defect profile behaves algebraically as a spinor, so that as a quasiparticle it can be likened to a fermion. We highlight that activity is a key ingredient to complete the mapping. This is because in passive nematic liquid crystals such Majorana-like excitations cost elastic energy, hence the associated spectrum is gapped, whereas in active nematics (18–22) they arise spontaneously due to the continuous energy input into the system, so that they may be viewed as gapless (or almost gapless) excitations.

Several condensed matter examples of Majorana quasiparticles have been found in the last two decades, for instance in the Kitaev chain made up of spinless electrons (11, 13), or in systems of magnetic skyrmions (23). However, previous realizations were always quantum, whereas the nematic analogue we propose is classical, which is appealing as it provides the tantalizing opportunity to observe quantum mechanical-like behavior at the mesoscale.

The physics of Majorana excitations (24) suggests that, if they can be stably produced in the lab, these quasiparticles can be braided and entangled in a controllable way, which may be useful for topological computing (25, 26). Indeed, liquid crystals have been recently proposed as an alternative medium to create new-generation computers and information processors (27); the mapping we propose in this work may provide further motivation to explore the potential of liquid crystals as topological computers. The Majorana mapping we discuss also sheds light, in turn, on the physics of active turbulence (28–30) in 3D, which has recently gained attention and has been experimentally realized in ref. 21. Specifically, by exploiting the analogy with quasiparticle phases in quantum condensed matter, we suggest that 3D active turbulence may be viewed as a topological phase where percolation of Majorana-like excitations creates a delocalized network of disclination lines, similar to the observation of delocalized topological Majorana modes in the Kitaev chain at the transition between the trivial and topological phase. This scenario is also reminiscent of other classical thermodynamic transitions, such as 2D melting, where the transition between the hexatic to the fluid phase is associated to defect unbinding and percolation of disclinations (31, 32).

## Mapping between Majorana Particles and Nematic Disclination Lines

### 1 + 1D Majorana Particles and Local Defect Profiles.

To begin with, we review the physics of Majorana spinors in 1+1D, following ref. 24. To construct them, it is customary to start from a Clifford algebra, which in 1+1D can be built from the following two matrices (or generators),

[1] $$\begin{array}{c} e_{1}=\left(\begin{array}{cc} -1 & 0\\ 0 & 1 \end{array}\right),\;e_{2}=\left(\begin{array}{cc} 0 & 1\\ 1 & 0 \end{array}\right). \end{array}$$

The algebra is such that the generators anticommute and have square equal to unity (24, 33),

[2] $$e_{1}^{2}=1,\;e_{2}^{2}=1,\;e_{1}e_{2}+e_{2}e_{1}=0.$$

Note that the associated Clifford algebra is called Cl(2,0) (34), as it has two generators ($e_{1}$ and $e_{2}$) squaring to +1 and no generator squaring to −1.

The Majorana equation is similar to the Dirac equation, except it describes a fermion which is equivalent to its antiparticle. It can be written as Dψ=0, where ψ is the spinor solution, and the Majorana operator D can be written in terms of Clifford algebra elements as follows,

[3] $$D=ie_{2}\frac{\partial }{\partial t}+ie_{2}e_{1}\frac{\partial }{\partial x}-ie_{1}m.$$

Let us define $\psi_{0}=e^{i(px-Et)}$, a (scalar) function associated with a free particle of mass m, momentum p, and energy E (where we have set the speed of light c=1). We call

[4] $$D\psi_{0}=\left[e_{2}\left(E-e_{1}p\right)-ie_{1}m\right]\psi_{0}\equiv \left(A+iB\right)\psi_{0}.$$

After some algebra, it can also be seen that A+iB is nilpotent, as ${\left(A+iB\right)}^{2}=E^{2}-p^{2}-m^{2}=0$ for our free particle. Therefore, the Clifford algebra element $\psi =\left(A+iB\right)\psi_{0}$ is such that Dψ=0, so that it provides a set of solutions for the Majorana equations in 1+1D. Specifically, the spinor solutions can be taken to be the column vectors in the matrix $\left(A+iB\right)\psi_{0}$, given by

[5] $$\psi =\left(\begin{array}{cc} -m\sin(\theta ) & \left(E-p)\cos(\theta \right)\\ (E+p)\cos(\theta ) & m\sin(\theta ) \end{array}\right),$$

where we have defined, for simplicity θ=px−Et. For a particle at rest (p=0), these solutions simplify to

[6] $$\psi =m\left(\begin{array}{cc} -\sin(\theta ) & \cos(\theta )\\ \cos(\theta ) & \sin(\theta ) \end{array}\right),$$

where now θ=−Et=−mt. In both cases, it can be verified that $\psi^{2}\propto 1$, which algebraically shows that the solution of the Majorana equation ψ is equivalent to its “antiparticle.”

Let us now analyze a disclination loop in a nematic liquid crystal. We consider a toroidal surface encircling the disclination line, which is topologically equivalent to $S_{1}\times S_{1}$. Following ref. 17, we call u the angle parameterizing the position along the smaller meridian $S_{1}$ circle, perpendicular to the disclination line, and v the angle determining the position along the larger longitudinal $S_{1}$ circle, tangential to the disclination. At an arbitrarily chosen origin on the loop, corresponding to v=0, the defect pattern can be described by a director field dependent on a single angle α(u),

[7] $$n=\left(\begin{array}{c} \cos(\alpha )\\ \sin(\alpha ) \end{array}\right).$$

The two components of n live on the plane locally perpendicular to the disclination tangent T.

The local director field pattern in Eq. **7** with α(u)=−1/2u corresponds to a triradius and can be seen as a Majorana-like quasiparticle at rest, as the solution is formally the same as the second column of Eq. **6**, where α(u) maps onto θ, so that u is the equivalent of time.^*^ Physically, the identification of a triradius as a Majorana-like quasiparticle is motivated by the well-known fact that its antiparticle, the comet-like defect with α(u)=+1/2u, is equivalent to it because one can be transformed into the other by rotating the director by π around an angle in the plane perpendicular to T (Fig. 1*B* and refs. 16 and 17). [Note that the comet-like solution would correspond to Eq. **6**, with θ→−θ.]

For the mapping between local defect profiles and Majorana quasiparticles to hold, we also need to show that the local defect profile transforms as a spinor. The representation in Eq. **7** as a 2×1 column vector is already suggestive of this fact. To understand the interpretation of the local defect profile as a spinor more in depth, we note that, in the two-dimensional Clifford algebra Cl(2,0) which we have been using, an element equivalent to the triradius is

[8] $$\begin{array}{cc} N_{0} & =\left(\begin{array}{cc} \cos(u/2) & \sin(u/2)\\ -\sin(u/2) & \cos(u/2) \end{array}\right)\\ & =\;\cos\left(u/2\right)1-\sin\left(u/2\right)e_{12}, \end{array}$$

where $e_{12}=e_{1}e_{2}$. Eq. **8** is nothing but Eq. **6** with the columns swapped (and θ→−u/2), and it can be readily recognized as a rotation matrix (with angle −u/2). The director profile corresponding to the −1/2 triradius defect can be identified with either of the column vectors in Eq. **8**, as these correspond to s=−1/2 defect profiles with initial angle α(u=0)=0 and π/2 respectively. The +1/2 defect director profile is instead given by Eq. **8** with u→−u. The element in Eq. **8** belongs to the even subalgebra generated by 1 and $e_{12}$, which is where spinors reside in Clifford algebra (39, 40),^†^ therefore showing that local defect profiles behave algebraically as spinors.

It is instructive to briefly discuss another possible construction of spinors in Cl(2,0), which is global, instead of local as in Eq. **8**. A triradius defect profile can be globally viewed as a −π rotation of the director field in the plane perpendicular to the disclination. Such a rotation can be represented again by Eq. **8**, but with u=π, which gives the bivector $-e_{12}$, that is also the generator of the so(2) Lie algebra. This simpler representation is useful as it renders the physical nature of the triradius defect profile as a Majorana quasiparticle especially clear. This is because: i) bivectors are naturally viewed as spinors (40); ii) the antiparticle of $-e_{12}$ is $e_{12}$, as multiplying the two gives the identity (because $-e_{12}^{2}=1$) and $e_{12}$ gives a global representation of the comet defect profile; iii) $\pm e_{12}$ are equivalent, as a spinor is defined up to a sign.

### Disclination Loops and the Importance of Activity.

While the local profile in Eq. **7** can be viewed as a Majorana-like quasiparticle *at rest*, we suggest that a closed disclination loop may be viewed as its counterpart with p≠0. It is well known that disclination loops in nematics can carry a charge Q, which is conserved modulo 2 (1, 4). For loops that are not pierced by other disclinations, there are only two possible topological classes. The first class is that of charged disclinations, which are topologically nontrivial, have Q=1, and are equivalent to a +1/2 or a −1/2 profile transported over the loop. These are qualitatively equivalent to a Majorana particle in 1+1D with finite momentum. The second class consists of uncharged loops with Q=0, which are topologically trivial and contain, for instance, one +1/2 profile and a −1/2 profile. Uncharged loops can shrink to 0 to leave a defect-free state. These loops are equivalent to a pair (or any even number) of Majorana quasiparticles, which annihilate with each other.

**Fig. 2.**
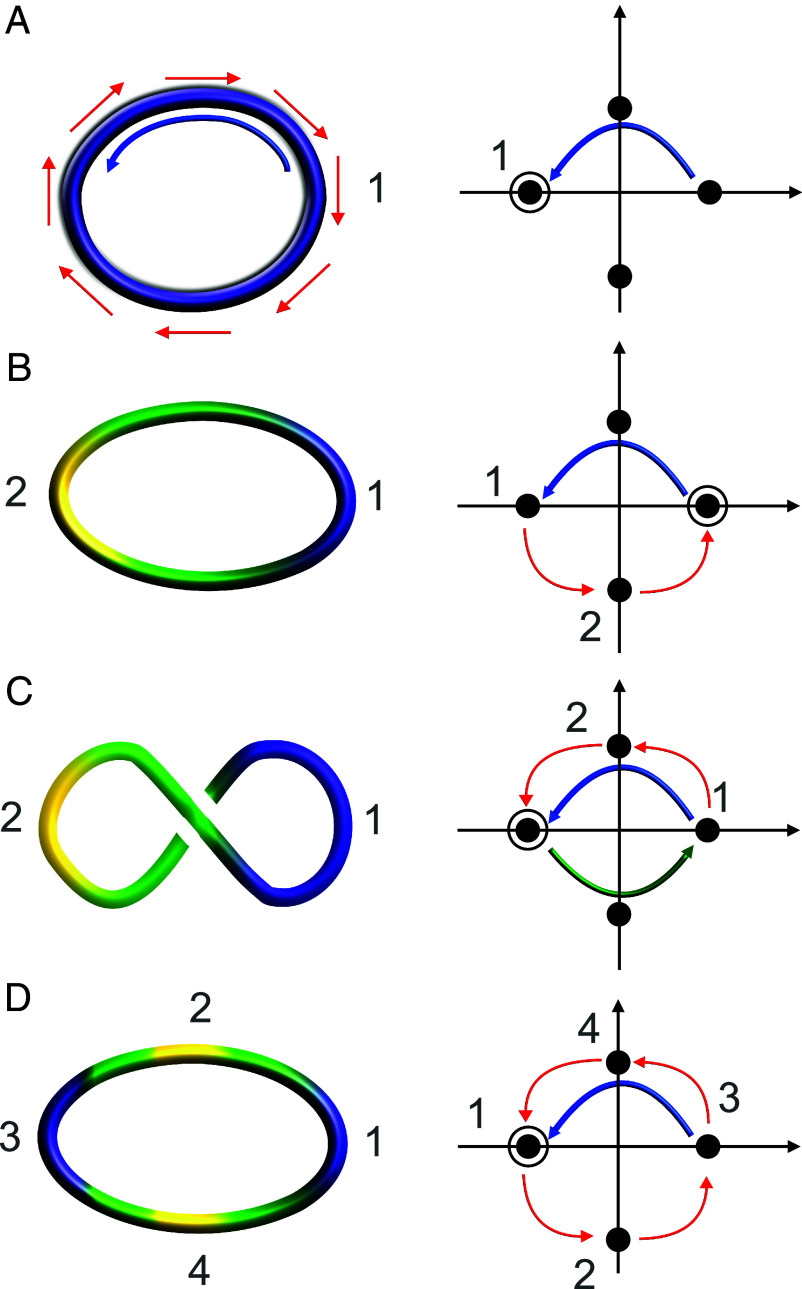
Sketches of topological patterns for disclination loops. (*A*) A Saturn ring with constant −1/2 (purple) profile (*Left*). This is topologically charged (*Right*); the blue line on the right corresponds to multiplication by −1, due to transport of the profile along the loop. (*B*) Topological pattern (*Left*) and charge diagram (*Right*) for +− loop, which is topologically uncharged. (*C*) An example of a writhed +− loop, here the writhing (green arrow) modifies the topology so that the loop is now charged. (*D*) A +−+− topologically charged loop. The pattern of the Ω vector is shown in red in the left sketch in (*A*), while the orientation of the tangent vector is shown in blue. The black circle denotes the end result of the charge calculation: if it is −1 (left point on the real axis), then the loop is charged; if it is +1, then the loop is uncharged. Note that we assume no self-linking (17), or twist of the defect profile along the disclination line: this assumption is justified by simulations for our active nematic patterns. On the other hand, twist disclinations can appear, and will feature in some of the examples in the main text.

For a single disclination, the charge Q can be analyzed, as in ref. 17, using quaternions describing either defect profile transitions, which consist of parity change (e.g., from +1/2 to −1/2) or profile rotations [associated with a nonzero self-linking number (17)]. To obtain a similar, but slightly simpler, picture, here we use the representation of a ±1/2 profile as $\pm e_{12}$ in Cl(2,0). Then, both a +1/2 and −1/2 defect profile, and a parity change from −1/2 to +1/2 profile and vice versa correspond to a left multiplication by $e_{12}$ and $-e_{12}$, respectively. By keeping track of the left multiplications, we can go through a disclination loop, to characterize the nature of the disclination behavior. The result of the chain of multiplications can be labeled by the elements of $Z_{4}$, namely ±1 or $\pm e_{12}$, which can be conveniently plotted in the complex plane if we make the identification of $e_{12}$ with the imaginary unit i (see the topological charge identification diagram in Fig. 2, right column).

The outcome of profile changes and rotations around the loop is given by the red arrows in Fig. 2 (right column) and can be visualized as a trajectory in $Z_{4}$, or as a series of multiplications by ±i on the complex plane. The result is an intrinsic topological index dependent on the sequence of local defect profiles along the loop. To arrive at the topological charge of the loop, we need to compose this index with an extrinsic one, describing the rotation of the defect profile caused by transport around the loop. The latter corresponds to a multiplication by −1 in Cl(2,0), or to a summation of 2 in the $Z_{4}$ ring. Similarly, if the loops writhe in 3d, then we essentially need to multiply by −1 for each crossing [or more precisely for each fractional unit of writhe (41)], or equivalently sum 2 in $Z_{4}$. This is because the transport of the defect profile along the loop and the self-crossings of the loop contribute to full rotations of the defect profile normal over the sphere, which modify the topological charge. If the result of the multiplication between the intrinsic (red arrows in Fig. 2) and the extrinsic topological index (blue and green arrows in Fig. 2) is 1, then the loop is uncharged, or Q=0; if it is −1, then the loop is charged, or Q=1. Take as an example the configuration in Fig. 2*D*. There are two back-and-forth changes between −1/2 and +1/2 corresponding to a full cycle in the complex plane denoted by the red arrows, the blue arrow then corresponds to a final half turn on the plane due to the profile transport around the loop: as a result, the loopis charged.

Disclination loops—whether charged (hence Majorana-like), or not—cost elastic energy, scaling as Kl, with K an elastic constant and l the disclination size. Given typical values of K in liquid crystals are ∼10 pN, a l∼1 μm loop costs ∼1.8 × 10^3^*k_B_T*, and is therefore a gapped excitation. Disclinations are therefore not seen in practice in a nematic liquid crystal close to its ground state. One promising way to obviate this issue is to use active liquid crystals (18–20) instead of passive ones. In active nematics, such as microtubule-kinesin or actomyosin mixtures, ATP-driven motors (kinesin or myosin) drive the system out of equilibrium and provide a source of nonthermal fluctuations with energy much larger than $k_{B}T$, leading to the spontaneous appearance of defects and disclination lines or loops in steady state (19). In other words, disclinations are gapless (or almost gapless) in active liquid crystals, and activity, which measures the density and strength of dipolar forces exerted by the molecular motors, can be thought of as an effective temperature, or inverse chemical potential. In what follows, we will therefore analyze various active nematic systems, and study the topological patterns of local defect profiles and disclination lines and loops in each of these, commenting on the appearance of Majorana-like features.

## Majorana Quasiparticles in 3D Active Nematics

### Structure and Dynamics of Majorana-Like Loops in Active Nematic Emulsions.

**Fig. 3.**
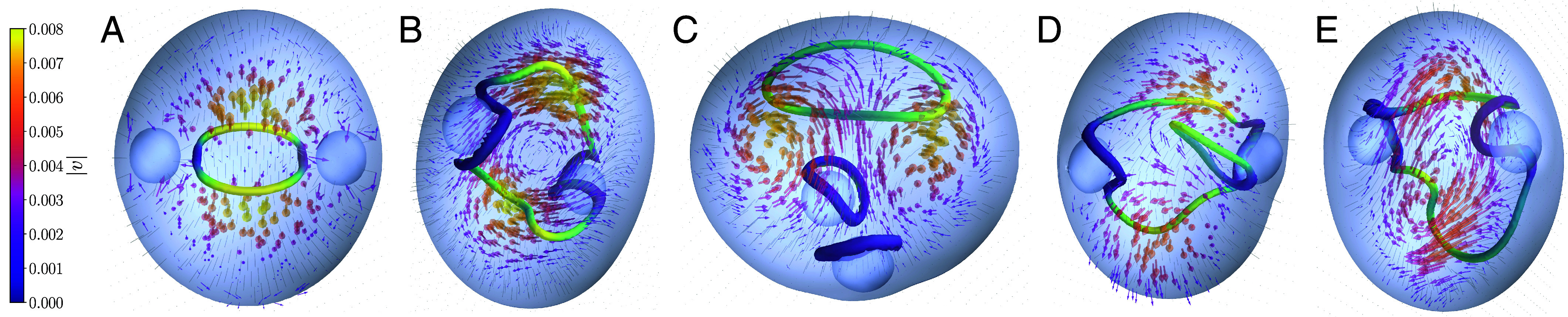
Director field pattern and disclination lines (colored according to the local cosβ value, as in Fig. 1), for an active nematic double emulsion, made up by inserting two isotropic passive droplets inside an active nematic droplet (38). The dimensionless value of the activity is $\sqrt{\zeta /K}R_{1}≃17.75$ (*A*) and $\sqrt{\zeta /K}R_{1}=35.5$ (*B*–*E*; see *Materials and Methods* for the full list of parameter values). A topologically charged (+−+−) loop can be seen for the lower activity (*A*), and for two of the snapshots at higher activity (*B* and *E*), where it is distorted in space. This loop corresponds to two changes back and forth from +1/2 to −1/2 local patterns. For the higher activity, states with three (*C*) or two (*D*) disclination loops are also possible; in all cases, the state is topologically nontrivial, so the sum of the loop charges equals 1mod2.

We start by considering an active double emulsion (*Materials and Methods* and ref. 38). This system is constituted by an active extensile nematic liquid crystal droplet (with radius $R_{1}$) which contains two smaller isotropic (passive and noncoalescing) droplets in its interior (with radius $R_{2}$, Fig. 3). All droplets are deformable and we consider normal anchoring on all surfaces. Because the internal droplets are equivalent to two topological point charges (“hedgehogs”), the overall geometry requires the director field pattern to be topologically nontrivial globally—i.e., it needs to include at least one charged, Majorana-like, disclination loop. We call ζ the activity and K the elastic constant of the active nematic liquid crystal.

Fig. 3*A* shows an example of the most likely topological pattern observed at intermediate activity, as the system starts to flow spontaneously due to activity (38). This pattern consists of a single rotating Majorana-like charged loop that bridges the two passive droplets. This Majorana-like loop, which as previously mentioned is topologically protected due to the emulsion geometry, features two parity transformations (17) back and forth from local +1/2 comet to −1/2 triradius profiles (Fig. 3*A*). Hence, we call this a +−+− loop; this is also the pattern shown in Fig. 2*D*. A Saturn ring embracing a colloid in a passive liquid crystal (42) would therefore be a − loop in this nomenclature. A single back-and-forth change (a +− loop) is instead equivalent to a topologically trivial loop (17). In terms of topological fermions, a +−+− loop can be viewed as a combination of three Majorana quasiparticles, which is topologically nontrivial. Each Majorana quasiparticle can then be more precisely identified with a 2π rotation of the Ω vector (or a π rotation in the charge diagram in Fig. 2*D*, right column). One of the rotations is the overall rotation of Ω around the loop contour, and the other two correspond to the two parity transformations.

While a charged loop is topologically required by the double emulsion geometry, activity extends the loop size such that it bridges the two isotropic droplets: for passive nematics, the defect profile would be similar but its size would be smaller (38). More importantly, in the passive limit the loop is static, whereas in our case, it is dynamic and rotates in steady state due to the active dipolar flow.

For larger activity, the spontaneous flow continuously injects energy into the system and leads to the continuous creation and disruption of states with multiple disclination loops with more complex topological patterns, which are not observed in passive nematic emulsions. Examples are shown in Fig. 3*B*–*E*, which gives four successive snapshots in a time series. At first, there is a single +−+− loop, which, while distorted has no writhe, hence is charged, and Majorana-like (Fig. 3*B*). This structure fragments into two Saturn rings, and another charged twist loop (Fig. 3*C*). At this point, there are therefore three separate Majorana-like loops. Later on, the disclinations rewire to yield a charged +−+− loop and an uncharged +− loop (Fig. 3*D*), and finally a single charged loop similar to the starting one (compare Fig. 3 *B* and *E*). The dynamics therefore show that pairs of Majorana-like loops (e.g., those in Fig. 3*C*) can indeed annihilate each other over time, and that activity leads to qualitatively different dynamics with respect to the purely relaxational one typical of the passive system. Notably, the droplet surfaces attract −1/2 local profiles, in analogy with colloidal particles which are surrounded by Saturn rings, which are everywhere equivalent to a −1/2 profile.

### Active Nematic Pipes and an Analogue of the Kitaev Chain.

To further explore the validity of the mapping between defect profiles and Majorana quasiparticles, we now consider a quasi-1D system where the nematic is confined along two directions, rather than inside a spherical droplet. Specifically, we consider an extensile nematic inside a long square parallelepiped (with $L_{y}=L_{z}$ the length of the short sides), with degenerate planar anchoring on the boundaries, and periodic boundary conditions along the long direction (43) (see *Materials and Methods* for the underlying free energy and computational details).

The motivation of the setup we consider is to seek a liquid crystalline analogue of the Kitaev chain, which is a 1D quantum condensed matter system with Majorana quasiparticles, whose physics we briefly review here for convenience (11, 13). In the Kitaev chain (11), a set of fermions with chemical potential μ hop along the lattice, and interact with each other via a superconducting pairing potential, creating Cooper pairs. With periodic boundary conditions, there is a transition between a strong-pairing phase, where the Cooper pair wavefunction decays exponentially, so that their size is small, to a weak-pairing phase, where the Cooper pair wavefunction decays more weakly, and tends to a constant for large separation (13), so that their size diverges. At the transition point, the Cooper pair delocalizes, and its size diverges. A $Z_{2}$ topological invariant, equivalent to the liquid crystalline topological charge Q, differentiates the two phases: it is zero in the strong-pairing, or topologically trivial, phase, and nonzero in the weak-pairing, or topological, phase (13).

With open boundary conditions, in the topological phase Majorana modes appear at the chain ends: within the Kitaev chain, these have zero energy, hence constitute the ground state of the system and are observable in practice. Their size is one lattice site for zero chemical potential, and it increases elsewhere in the topological phase, diverging at the transition (13).

We first consider cases where the passive limit (ζ=0) is a nematic liquid crystal. In this case, the ground state is defect-free, which, unlike the active emulsion case, is compatible with the quasi-1D periodic geometry we consider. Therefore, without activity Majorana loops cost too much free energy, and are not observed.

**Fig. 4.**
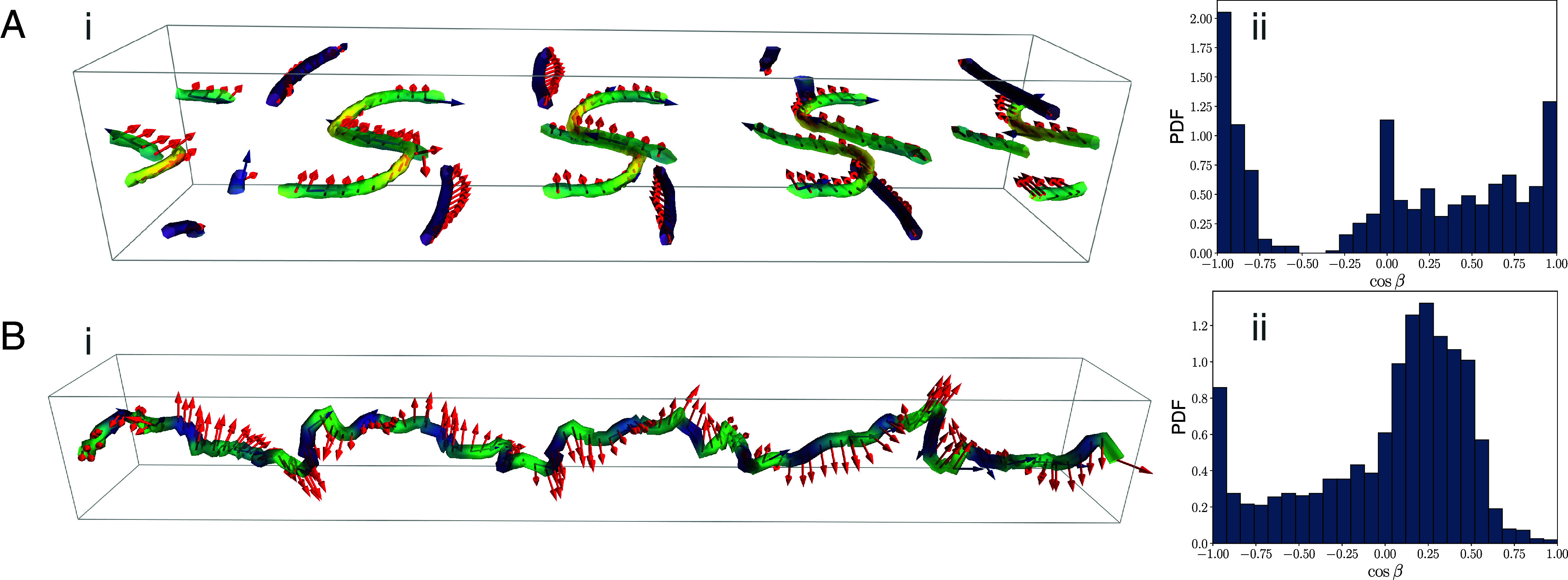
(*A*) i) Director field pattern and disclination lines (colored according to the local cosβ value, as in Fig. 1), for the vortex lattice first described in ref. 43. ii) Corresponding histogram of local values of cosβ, showing the abundance of ±1/2 Majorana-like local patterns. (*B*) i) Director field pattern and disclination lines (colored according to the local cosβ value) for the double helix state of (43). ii) Corresponding histogram of local values of cosβ.

If the activity is sufficiently increased ($\sqrt{\zeta /K}L_{y,z}≃22$; see *Materials and Methods* for full parameter list), a “vortex lattice” spontaneously appears (43), with defects in steady state (Fig. 4*A*) accompanied by active flow. At early times, the emerging defects appear as uncharged disclination loops (*SI Appendix*, Fig. S1). Later, charged disclination arcs of finite size stick to the boundary, corresponding to Majorana quasiparticles. The local value of cosβ=T·Ω, calculated from the disclination density tensor (*Materials and Methods* and Eq. **22**), peaks around −1 and +1, corresponding to local triradius and comet profiles respectively. The triradius profiles (cosβ=−1) localize at corners, while the comet profiles (cosβ=+1) most commonly stick to opposite walls and are stretched by the flow (Fig. 4*A*). This localization of defects close to surfaces is reminiscent of Majorana excitations found deep in the topological phase of the Kitaev chain, where, as reviewed above, they have finite size and localize at the ends of the chain (13).

For the same value of the activity, a different structure emerges, for weak anchoring and in the parameter regime where the liquid crystal is in the isotropic phase for ζ=0, but for sufficiently large ζ acquires activity-driven orientational nematic order due to shear associated with spontaneous flow (see *Materials and Methods* for full parameter list). This is the “double helix” state, which was first observed in ref. 43, and which is analyzed topologically in Fig. 4*B*. Here, the activity is sufficiently large ($\sqrt{\zeta /K}L_{y,z}≃25$) to offset the thermodynamic cost of defects, such that they emerge spontaneously. In the double helix state, the system self-organizes to create a helical flow state with the main velocity component along the direction of the channel and with a defined handedness arising through spontaneous chiral symmetry breaking. This structure is associated with an extended chiral disclination line with the same handedness, which can be viewed as a gapless, or zero-energy, excitation (Fig. 4*B*; left-handed instance). This is reminiscent of a delocalized topological state in the Kitaev chain, which in the latter system is observed, as previously reviewed, right at the transition between the trivial and topological phases. The global topological charge of the extended disclination is zero, as the pattern is characterized by an even winding of the rotation vector Ω around the tangent to the disclination line. Note that, because its global topological charge is zero, this disclination line is equivalent to an even number of Majorana quasiparticles.

### Active Turbulence as a Topological Phase with Delocalized Quasiparticles.

**Fig. 5.**
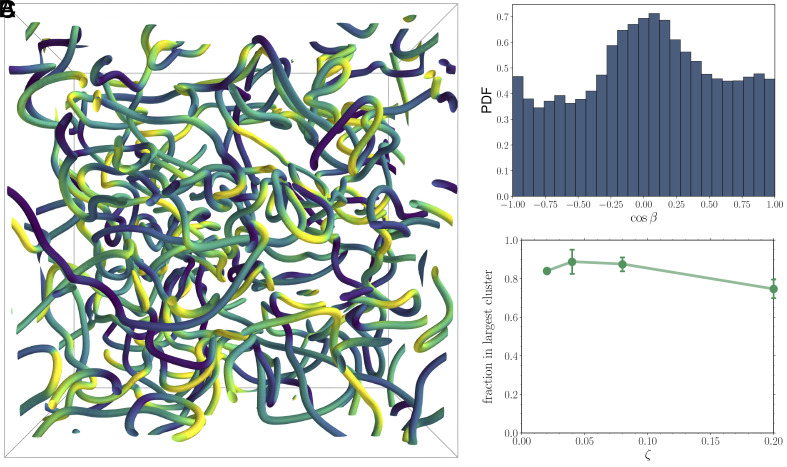
(*A*) Snapshot of an active contractile nematic in bulk (parameter set given in *Materials and Methods*). Disclination lines are colored according to the local cosβ value. (*B*) Corresponding histogram of local values of cosβ. (*C*) A plot of the fraction of the disclination network in the largest connected defect cluster as a function of ζ (complete parameter set given in *Materials and Methods*). Defects are identified here as regions in space with the order parameter smaller than 0.15.

In both the examples of Figs. 3 and 4, we considered a confined active nematic fluid. It is also of interest to study the behavior of disclinations as quasiparticles in the bulk, without confinement. We focus on contractile activity in this case, to complement the extensile case studied under confinement. Such a contractile system could be experimentally realized with uniform actomyosin mixtures in 3D. With periodic boundary conditions, the total charge of loops must be equal to 0 modulo 2. Similar to the channel case, the system is therefore defect-free for zero or low activity ($\sqrt{\zeta /K}L\overset{<}{∼}15$, where L is the simulation box size); whereas, for larger activity, we observe active turbulence, with a network of disclinations and loops (Fig. 5*A*).

By analyzing the topology and morphology of the disclination networks formed at different values of contractile activity ζ, the following main observations can be made. First, the local topological patterns (identified via the cosβ values along disclination lines) are different from those of extensile nematics, where the local defect structure usually corresponds to a radial or tangential twist profile corresponding to cosβ=0 (21, 22). In the contractile case studied here, local profiles include more often triradii and comet-like defects (Fig. 5*B*), which in our mapping correspond to more pronounced Majorana-like local features. Second, virtually all the loops we have analyzed are instead topologically trivial globally. This result points to the key role played by boundaries (absent in this example) in stabilizing topologically charged loops.

A third result is that an ensemble of short loops (corresponding to localized excitations, or quasiparticles) coexist with one or few very large percolating disclinations which wrap the system (Fig. 5*C* and *SI Appendix*, Fig. S2). This is similar to what was observed in recent simulations of extensile active turbulence in 3D (44), where it was additionally found that the distribution of disclination length is power-law distributed. The mapping between disclination lines and topological quasiparticles is of use here to explain these observations. The long wrapping disclination lines are an example of delocalized topological excitations, which we have seen appear in the double helix phase in active nematic channels, as well as in the weak-pairing phase of the Kitaev chain (13). This analogy suggests that active turbulence can be thought of as a topological phase, where disclinations percolate at the transition between defect-free and turbulent phases. This scenario is also reminiscent of other classical liquid crystal transitions, most notably the hexatic-fluid transition at which structural defects percolate in 2D (31, 32).

## Discussion and Conclusions

In summary, here we have shown that local defect profiles and topologically charged disclination loops in a 3D active nematic liquid crystal can be mapped to Majorana quasiparticles. This mapping—which is the main result of our work—builds from the well-known equivalence between −1/2 and +1/2 local profiles in 3D. Therefore, a 2D defect pattern can be smoothly transformed into its “antidefect,” which is a defining feature of a Majorana particle. The mapping also requires proving that the defect profile transforms as a spinor, which can be done by using an algebraic representation of defect profiles within 2D Clifford algebras. More precisely, we map a topologically trivial uncharged disclination loop to a pair of (annihilating) Majorana particles, while a topologically nontrivial charged loop maps to a Majorana quasiparticle in 1+1D. Activity is an important ingredient to observe these Majorana loops in a steady state, otherwise (in the absence of boundaries) they cost too much free energy and cannot be observed.

We have shown that the analogy runs deeper, as we can qualitatively recreate a Kitaev chain in a confined quasi-1D active nematic fluid. Here, the activity provides a parameter similar to chemical potential in the Kitaev chain, as it can be tuned to trigger a transition between a trivial (passive) phase and a topological (active nematic) phase. In the trivial phase, at low activity, Majorana excitations are gapped and hence absent. In the topological phase, at large activity, Majorana-like disclinations emerge spontaneously, as arcs absorbed onto the surface. Additionally, at higher effective temperatures (and weaker anchoring) an extended helical disclination line arises (Fig. 4), reminiscent of delocalized topological modes found in the Kitaev chain close to the transition between the trivial and topological (or strong-pairing and weak-pairing) phases.

On the one hand, the mapping between Majorana particles and disclination loops links distant parts of physics and is of interest because of the rarity of Majorana spinors, which are absent in the current version of the standard model and are challenging to experimentally observe in quantum condensed matter. On the other hand, we suggest this mapping can be of practical use as well. First, the Clifford algebra formalism we used to map disclinations to spinors can be extended to provide a simple understanding of the topological charge of a generic disclination loop in nematics (Fig. 2). Second, one can use active liquid crystalline emulsions to spontaneously create Majorana-like disclination loops (Fig. 3) which could be studied at much larger lengths and time scales which is possible for quantum systems. Third, the analogy between nematic loops and quasiparticles suggests an intriguing interpretation of active turbulence as a topological phase (Fig. 5). This interpretation has the virtue of naturally explaining why, in the active turbulent phase, there are system-wrapping disclination lines that coexist with small loops. As for the double helix state observed in confinement, these are delocalized topological modes, which are expected to occur within a topological phase. In this scenario, active turbulence can be likened to passive topological phases such as the blue phases of cholesterics (2), and the transition between defect-free phase and active turbulence to the hexatic-fluid transition where defects percolate in 2D.

We hope that the mapping between active disclination loops and Majorana quasiparticles we have discussed will stimulate further work in different directions. First, we suggest that similar mapping between topological defects and quasiparticles should exist in biaxial nematics and cholesterics (45, 46), as chirality further stabilizes charged disclinations even in passive systems (47). It would be interesting to characterize these defects as quasiparticles and investigate which type of spinor represents them. It would also be desirable to map out the dispersion relation which characterizes active nematic quasiparticles in our cases, as this relation plays a role analogous to that of the Majorana equation in linking quasiparticle frequency and momentum. Second, another avenue to explore, which has not been studied here, is the braiding of disclinations. Biaxial nematics and cholesterics allow for defect braiding (4, 17), while activity brings about the possibility that vortex tubes interact with disclination lines in yet-to-be-determined ways, generalizing their 2D coupled dynamics characterized in ref. 37. Finally, it would appear natural to apply the ideas discussed here within the context of nbits introduced in ref. 27, to engineer new topological phases of active materials which can contribute to topological computing.

## Materials and Methods

### Hydrodynamic Equations of Motion.

We outline the general hydrodynamic equations we solved to obtain results for the three considered in the main text: i) active emulsion (Fig. 3); ii) active nematic channel (Fig. 4); and iii) active turbulence (Fig. 5). System-specific details are given later for each case individually.

We describe the physics of an active nematic fluid by considering two coarse-grained fields: i) the fluid velocity v(r,t), and ii) the tensorial order parameter Q(r,t) (48) accounting for the ordering of a liquid crystal made of rod-like molecules.

The properties of the system in the passive limit are encoded by a free energy F, whose density f depends on the order parameter Q. A form of the free energy density which is appropriate to describe a nematic liquid crystal is

[9] $$\begin{array}{cc} f_{\mathrm{LC}} & =\frac{A_{0}}{2}\left(1-\frac{\gamma }{3}\right)Q_{\alpha \beta }^{2}-\frac{A_{0}\gamma }{3}Q_{\alpha \beta }Q_{\beta \gamma }Q_{\gamma \alpha }\\ & +\;\frac{A_{0}\gamma }{4}{\left(Q_{\alpha \beta }^{2}\right)}^{2}+\frac{K}{2}{\left(\partial_{\gamma }Q_{\alpha \beta }\right)}^{2}, \end{array}$$

where Greek indices denote the Cartesian components of tensors and summation over repeated indices is implied. The first three terms on the right-hand side of Eq. **9** constitute the bulk free energy density and are the first terms in an expansion over powers of the Q tensor, where $A_{0}$ is a positive constant and γ a parameter controlling the transition between isotropic and nematic phase at $\gamma <\gamma_{c}=2.7$ and $\gamma >\gamma_{c}$, respectively. The energetic cost due to liquid crystal distortions is controlled by the gradients of Q and is proportional to K, the elastic constant.

The time evolution of the Q tensor is governed by the Beris-Edwards equation (49)

[10] $$\left(\partial_{t}+v\cdot \nabla \right)Q-S\left(W,Q\right)=\Gamma H,$$

where Γ is a collective rotational diffusion constant and H is the molecular field, which is given by

[11] $$H=-\frac{\delta F}{\delta Q}+\left(\frac{I}{3}\right)\;\text{Tr}\frac{\delta F}{\delta Q},$$

with I the identity matrix. The first term on the left-hand side of Eq. **10** represents the material derivative accounting for the time dependence of a quantity advected by the fluid velocity v. The second term accounts for the rotation and stretching of the rod-like molecules of the liquid crystals due to flow gradients (49). This is given by

[12] $$\begin{array}{cc} S(W,Q) & =(\xi D+\omega )\cdot (Q+I/3)\\ & +\;(Q+I/3)\cdot (\xi D-\omega )\\ & -\;2\xi (Q+I/3)\;\text{Tr}(Q\cdot W). \end{array}$$

Here, Tr denotes the tensorial trace, while $D=(W+W^{T})/2$ and $\omega =(W-W^{T})/2$ are the symmetric and antisymmetric part of the velocity gradient tensor $W_{\alpha \beta }=\partial_{\beta }v_{\alpha }$. The parameter ξ determines whether the liquid crystal is flow-aligning or flow-tumbling.

The fluid velocity v evolves according to the Navier–Stokes equation,

[13] $$\rho \left(\partial_{t}v+v\cdot \nabla v\right)=-\nabla p+\nabla \cdot \left(\sigma^{\mathrm{visc}}+\sigma^{\mathrm{lc}}+\sigma^{\mathrm{act}}\right),$$

complemented by the incompressibility condition ∇·v=0. In Eq. **13**, ρ is the fluid density and p is the hydrodynamic pressure. The stress σ is broken up into three terms. The viscous contribution, $\sigma^{\mathrm{visc}}$, is given by

[14] $$\sigma_{\alpha \beta }^{\mathrm{visc}}=\eta^{\mathrm{visc}}\left(\partial_{\alpha }v_{\beta }+\partial_{\beta }v_{\alpha }\right),$$

where $\eta^{\mathrm{visc}}$ is the shear viscosity of the fluid. The elastic stress due to liquid crystal deformations, $\sigma^{\mathrm{lc}}$, reads

[15] $$\begin{array}{cc} \sigma_{\alpha \beta }^{\mathrm{lc}} & =-\xi H_{\alpha \gamma }\left(Q_{\gamma \beta }+\frac{1}{3}\delta_{\gamma \beta }\right)-\xi \left(Q_{\alpha \gamma }+\frac{1}{3}\delta_{\alpha \gamma }\right)H_{\gamma \beta }\\ & +\;2\xi \left(Q_{\alpha \beta }+\frac{1}{3}\delta_{\alpha \beta }\right)Q_{\gamma \mu }H_{\gamma \mu }+Q_{\alpha \gamma }H_{\gamma \beta }-H_{\alpha \gamma }Q_{\gamma \beta }\\ & -\;\partial_{\alpha }Q_{\gamma \mu }\frac{\partial f}{\partial (\partial_{\beta }Q_{\gamma \mu })}. \end{array}$$

The third term, $\sigma^{\mathrm{act}}$, is the active stress given by (19)

[16] $$\sigma_{\alpha \beta }^{\mathrm{act}}=-\zeta Q_{\alpha \beta },$$

where the activity parameter ζ is positive for extensile materials and negative for contractile ones.

There are two key dimensionless parameters in all systems we considered. One is the reduced activity number $\tilde{A}$, which is the ratio between a typical lengthscale l (droplet radius or channel width or system size, according to the system of interest) and the active lengthscale $l_{a}=\sqrt{K/\zeta }$ (19). The other is the reduced temperature τ=27(1−γ/3)/γ, which determines whether the system in the passive phase is isotropic (τ>1) or nematic (τ<1). A third dimensionless parameter determines the strength of anchoring at boundaries in systems with confinement.

### Active Emulsion.

To describe an active emulsion, a set of passive scalar phase fields $\phi_{i}\left(r,t\right),i=1,2,3$ is required to capture the density of each droplet in addition to the velocity field and the Q tensor. The active droplet in Fig. 3 is associated with the phase field $\phi_{1}$, whereas the two passive droplets are with $\phi_{2,3}$.

The free energy density in Eq. **9** also needs to be complemented by an additional term to describe the behavior of the phase fields; this reads,

[17] $$\begin{array}{cc} f_{\phi } & =\frac{a}{4}\sum_{i=1}^{3}\left[\phi_{i}^{2}{\left(\phi_{i}-\phi_{0}\right)}^{2}\right]+\frac{k}{2}\sum_{i=1}^{3}{\left(\nabla \phi_{i}\right)}^{2}\\ & +\;\epsilon_{\phi }\sum_{i\neq j;i,j=1}^{3}\phi_{i}^{2}\phi_{j}^{2}+W_{\phi }\sum_{i=1}^{3}\left[\partial_{\alpha }\phi_{i}Q_{\alpha \beta }\partial_{\beta }\phi_{i}\right]. \end{array}$$

The first term in Eq. **17**, multiplied by the positive constant a, represents a double-well potential which ensures the existence of two coexisting minima at $\phi_{1,2,3}=\phi_{0}=2$ (this represents the inside of droplets 2 and 3 and the outside of droplet 1) and $\phi_{1,2,3}=0$ (inside of droplet 1 and outside the droplets 2 and 3). The second term, multiplied by the elastic constant k, controls the interfacial energy. The constants a and k together determine the surface tension $\sigma =\sqrt{8ak/9}$ and the interface thickness $\xi_{\phi }=\sqrt{2k/a}$ of the droplets. The third contribution is a soft-core repulsion, whose magnitude is controlled by the positive constant $\epsilon_{\phi }$. In $f_{\mathrm{LC}}$ (Eq. **9**), the coefficient γ is now a function of the phase fields. Following previous works (50), we set $\gamma =\gamma_{0}+\gamma_{s}\sum_{i=1}^{3}\phi_{i}$, where $\gamma_{0}$ and $\gamma_{s}$ control the boundary of the coexistence region. Note that γ depends on the sum of $\phi_{i}$, since the liquid crystal is confined solely within the layer where $\sum_{i=1}^{3}\phi_{i}=0$. The anchoring of the director at the droplet interface is described by the last term, where $W_{\phi }$ is the anchoring strength ($W_{\phi }<0$ corresponds to normal, or homeotropic, anchoring at droplet surfaces).

The dynamics of the scalar fields $\phi_{i}$ (i=1,2,3) obeys a Cahn–Hilliard equation

[18] $$\partial_{t}\phi_{i}+v\cdot \nabla \phi_{i}=M\nabla^{2}\mu_{i},$$

where M is the mobility and $\mu_{i}=\frac{\delta F}{\delta \phi_{i}}$ is the chemical potential.

A further contribution to the stress stems from interfacial stress and is given by

[19] $$\sigma_{\alpha \beta }^{\mathrm{int}}=\sum_{i=1}^{3}\left[\left(f-\phi_{i}\frac{\delta F}{\delta \phi_{i}}\right)\delta_{\alpha \beta }-\frac{\partial f}{\partial (\partial_{\beta }\phi_{i})}\partial_{\alpha }\phi_{i}\right].$$

As in previous works (50), we used a 3D hybrid lattice Boltzmann (LB) method which solves Eqs. **10** and **18** via a finite difference scheme while Eq. **13** was solved by a lattice Boltzmann approach. Parameters used to generate the configuration shown in Fig. 3 are σ≃0.035 $\xi_{\phi }≃5.29$, M=0.1, $R_{1}/l_{a}≃17.75$ (Fig. 3*A*) and $R_{1}/l_{a}≃35.5$ (Fig. 3*B*–*E*; note for all panels $R_{1}=32$ is the initial radius of the active droplet, whereas $R_{2}=R_{3}=8$ are the initial radii of the isotropic cores), dimensionless anchoring strength $W_{\phi }R/\left(K\xi_{\phi }\right)≃-4.65$, $A_{0}=0.12$, $\gamma_{0}=2.85$, $\gamma_{s}=-0.25$ so that in the active nematic region τ≃0.47.

### Active Channel.

Hybrid LB active channel simulations (Fig. 4) were confined by impermeable no-slip planar walls. The free energy density in Eq. **9** is complemented by an anchoring free energy term with minima at $Q=S_{0}\left(\nu ⊗\nu -\frac{1}{3}1\right)$, with $S_{0}$ the preferred degree of surface order and ν an arbitrary orientation parallel to the surface. This anchoring free energy reads (51)

[20] $$\begin{array}{cc} F_{\mathrm{anch}} & =\int dS[W_{1}\left(\tilde{Q}-{\tilde{Q}}^{⊥}\right):\left(\tilde{Q}-{\tilde{Q}}^{⊥}\right)\\ & +\;W_{2}\left(\tilde{Q}:\tilde{Q}-S_{0}^{2}\right)]. \end{array}$$

The first term imposes *alignment* on the tangent plane with an energy cost $W_{1}$. Here, $\tilde{Q}=Q+\frac{1}{3}S_{0}1$ and ${\tilde{Q}}^{⊥}=P\cdot \tilde{Q}\cdot P$ in terms of the projection operator P on the plane perpendicular to the surface normal. The second term sets the *degree* of order ($S_{0}$) on the plane, with energy cost $W_{2}$. In simulations analyzed here, $W_{1}=W_{2}$.

The active nematic fluid is confined within a parallelepiped of size $L_{x}\times L_{y}\times L_{z}$. For both the states in Fig. 4, ρ=1, $\eta^{\mathrm{visc}}=4/3$, Γ=0.3375, ξ=1, $L_{y}=L_{z}=25$. Additionally, for the vortex lattice (Fig. 4*A*), $L_{x}=130$, $A_{0}=1$, τ=0 (γ=3), $\sqrt{\zeta /K}L_{y,z}=22$, $W_{1,2}L_{y,z}/K=250$. For the double helical state (Fig. 4B), $L_{x}=200$, $A_{0}=0.1$, τ≃1.120 (γ=2.668), $\sqrt{|\zeta |/K}L_{y,z}=25$, and dimensionless anchoring strength at the walls $W_{1,2}L_{y,z}/K=1.5$.

### Active Turbulence.

Active turbulence simulations (Fig. 5) are based on Eq. **9**, and were performed in a cubic box of size $L=256\sqrt{K/A_{0}}$ with periodic boundary conditions. Simulations were solved numerically with an in-house MPI-parallel fully dealiased pseudospectral code developed within the Dedalus framework (52). All fields are represented by a triple-Fourier spectral decomposition with the spectral resolution being given by the number of modes in each direction $\left(N_{x},N_{y},N_{z}\right)$. Production runs are carried out using $N_{x}=N_{y}=N_{z}=256$ comprising approximately $1.5\times 10^{8}$ degrees of freedom. Temporal discretization employs the semi-implicit backward differentiation scheme of order four (53) with a time step of 0.01. All simulations were evolved for at least $10^{4}$ time units in a statistically steady state, where time is measured in units of ${\left(\Gamma A_{0}\right)}^{-1}$. Parameters corresponding to Fig. 5 are Γ=0.3, ξ=0.7, $A_{0}=1.5$, τ=0 (γ=3), K=0.06, ζ=−0.08 (Fig. 5 *A* and *B*) and ζ∈{−0.02,−0.2} (Fig. 5*C*). Therefore, the ratio between the system size and the active lengthscale is ≃59.12 in Fig. 5 *A* and *B*, and between 29.56 and 93.48 in Fig. 5*C*.

### Defect Analysis.

Within the theory of Majorana particles and bound states, the Majorana polarization and Pfaffian charge density (54) provide useful quantities to identify topologically nontrivial states associated with Majorana physics. For our nematic counterpart, a qualitatively analogous quantity is the disclination line tensor (36), which gives a way to visualize the local defect profile of a liquid crystalline pattern in a simple way (37), determining whether the profile on a disclination segment is triradius-like, comet-like, or twist-like (Fig. 1). The tensor is constructed from derivatives of the Q-tensor,

[21] $$D_{\mathrm{ij}}=\epsilon_{i\mu \nu }\epsilon_{\mathrm{jlk}}\partial_{l}Q_{\mu \alpha }\partial_{k}Q_{\nu \alpha },$$

where i,j,k,α,μ,ν are tensor indices with applied summation convention. This form is useful due to the interpretation as the dyad composing of the local line tangent T and the rotation vector Ω

[22] $$D_{\mathrm{ij}}=s\left(r\right)\Omega_{i}T_{j}.$$

where s(r) is a positive scalar field that is maximum at the disclination core. Defects are identified as isosurfaces of $s\left(r\right)=s_{\mathrm{cut}}$. For the double emulsion $s_{\mathrm{cut}}=0.033$, double helix $s_{\mathrm{cut}}=0.023$, vortex lattice $s_{\mathrm{cut}}=0.1$ and active turbulence $s_{\mathrm{cut}}=0.01$. For Fig. 3 (Majorana-like loops) and Fig. 4*B*, i (double helix configuration), topological patterns are visualized as a tube that connects an ordered sequence of points along the defect loop.

Extracting Ω and T use the methods outlined in ref. 36, ensuring that the vectors are continuous along the loop and have the correct relative sign, set by sgn(Ω·T)=sgn(Tr(D)). Visualizations of disclinations and disclination networks use the Mayavi library (55).

## Supplementary Material

Appendix 01 (PDF)

## Data Availability

All study data are included in the article and/or *SI Appendix*.

## Footnotes

^*^We note an early proposal of a link between a (different) passive nematic defect line and Majorana particles was put forward in ref. 35.

^†^Indeed, it can be shown that, within Clifford algebras, spinors may be mathematically represented either as minimal left ideals or elements of the even-graded subalgebra (40), essentially as these have the same number of degrees of freedom—which is 2 for Cl(2,0).
