# Supplementary material for: Majorana quasiparticles and topological phases in 3D active nematics
Source: Proc Natl Acad Sci U S A. 2024 Dec 19;121(52):e2405304121. doi: 10.1073/pnas.2405304121 (PMC11670186; doi:10.1073/pnas.2405304121)
Supplement: Supplementary file 1 — Appendix 01 (PDF) [file pnas.2405304121.sapp.pdf]

Majorana quasiparticles and topological phases in 3D active nematics: Supporting Information

Louise C. Head,[1] Giuseppe Negro,[2] Livio N. Carenza,[3] Nathan Johnson,[1] Ryan R. Keogh,[1] Giuseppe Gonnella,[2] Alexander Morozov,[1] Enzo Orlandini,[4] Tyler N. Shendruk,[1] Adriano Tiribocchi,[5] and Davide Marenduzzo[1]

[1] *SUPA, School of Physics and Astronomy, University of Edinburgh, Peter Guthrie Tait Road, Edinburgh, EH9 3FD, UK*

[2] *Dipartimento di Fisica, Università degli Studi di Bari and INFN, Sezione di Bari, via Amendola 173, Bari, I-70126, Italy*

[3] *Faculty CS Physics, Koc University, Rumelifeneri Yolu 34450 Sariyer, Istanbul, Turkey*

[4] *Department of Physics and Astronomy, University of Padova and INFN, Sezione Padova, Via Marzolo 8, I-35131 Padova, Italy*

[5] *Istituto per le Applicazioni del Calcolo, Consiglio Nazionale delle Ricerche, via dei Taurini 19, Roma, 00185, Italy*

SUPPLEMENTARY FIGURES

Figure S1

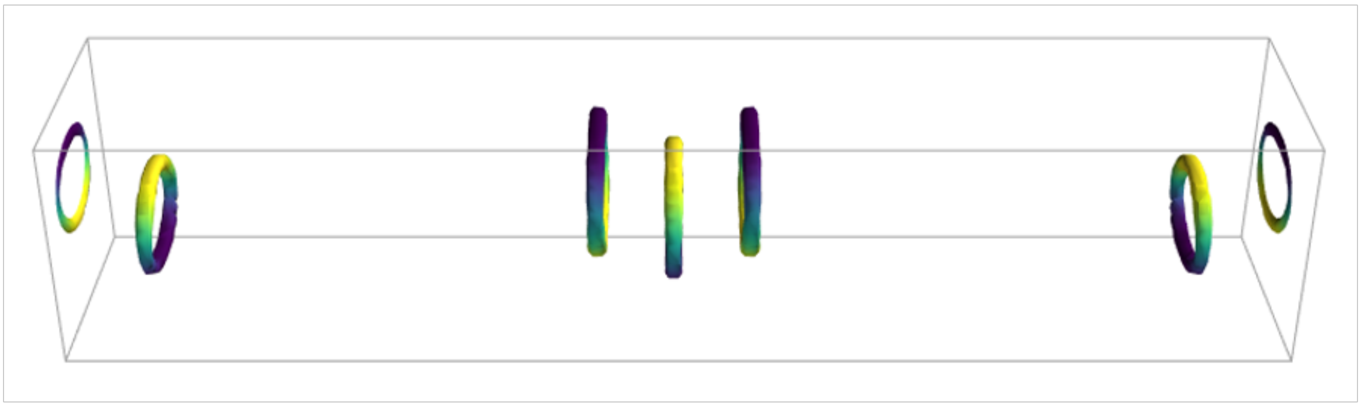

Fig. S1 Vortex lattice topology at early times. Patterns of disclination loops (coloured according to the local $\cos\beta$ value, as in Fig. 1 of the main text), for the vortex lattice (whose steady state configuration is shown in Fig. 4Ai).

Figure S2

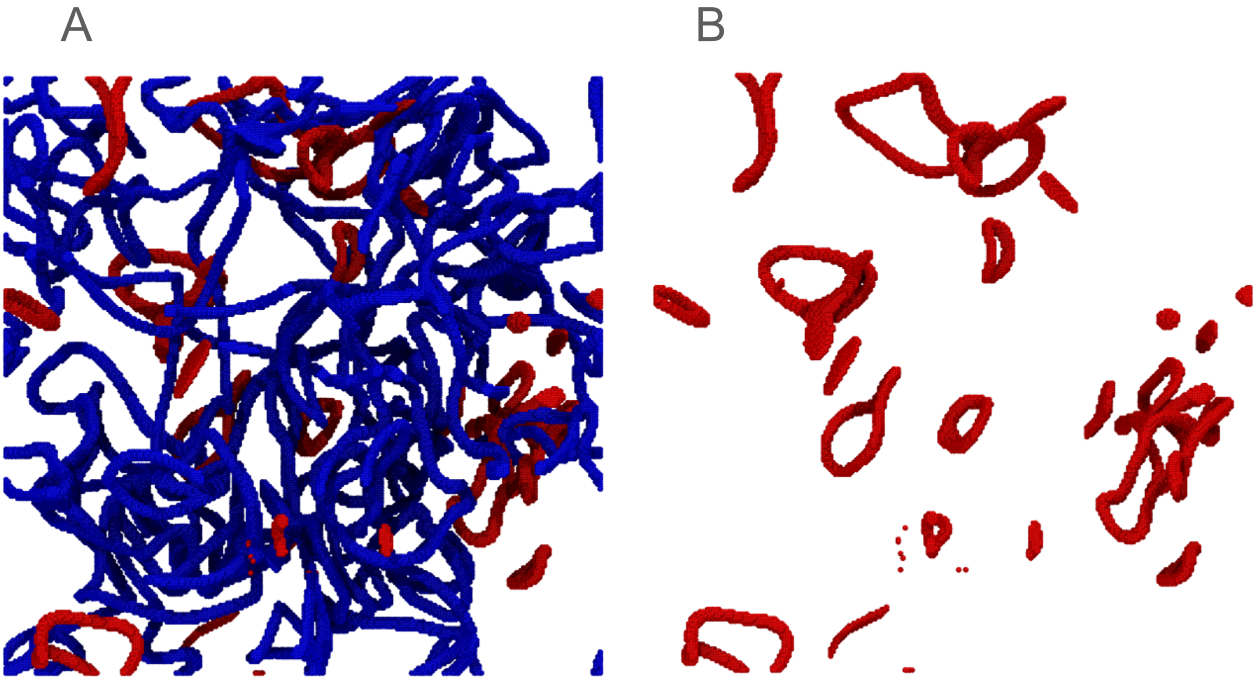

Fig. S2 Disclination line patterns in active turbulence. A. Disclination line patterns coloured according to whether a defect (order parameter smaller than 0.18) is in the largest cluster (blue) or not (red). Parameters correspond to those of Fig. 5C, for $\zeta = -0.08$. B. Same as A but without the largest connected component.
